# Experimental and Computational Insights into SERS-Based Ultrasensitive Detection of the Pesticide Fenazaquin: Towards Food Safety and Environmental Analysis

**DOI:** 10.3390/molecules31214009

**Published:** 2026-11-06

**Authors:** Oumaima Douass, Ibrahim Saadouni, Bousselham Samoudi, Ana Crespo, Santiago Sanchez-Cortes

**Affiliations:** 1 Intelligent System Design Laboratory, Optics, Material and Systems Team, Faculty of Sciences, Abdelmalek Essâadi University, B.P. 2121, M’ Hannech II, Tétouan 93030, Morocco; oumaima.douass@etu.uae.ac.ma (O.D.);; 2 Instituto de Estructura de la Materia, Consejo Superior de Investigaciones Científicas (IEM-CSIC), Serrano 121, 28006 Madrid, Spain; a.crespo.i@csic.es

**Keywords:** Ag nanoparticules, SERS, fenazaquin, charge-transfer, DFT, Monte Carlo

## Abstract

In this work, SERS is employed as an effective detection technique for Fenazaquin (FEN) due to its notable specificity and sensitivity. The investigation involves a comprehensive analysis using UV–Vis spectroscopy, Raman spectroscopy and SERS on solutions containing varying concentrations of FEN. Different experimental conditions were examined to optimize the detection process, with Raman spectra acquired for FEN powder. SERS analyses were conducted across a concentration range of 9.9 × 10^−8^ to 1.9 × 10^−6^ mol/L. The selection of suitable substrates is crucial for optimizing SERS performance. Herein, the SERS signals of FEN were studied on Ag nanoparticles reduced by hydroxylamine (AgSH), with aggregation induced by KNO_3_ salt. To support the experimental data, the molecular structure and theoretical Raman spectra of FEN were calculated using Density Functional Theory (DFT). Furthermore, to gain insights into the interaction mechanisms between FEN and the Ag substrate, Adsorption Locator Monte Carlo simulations were employed. These simulations predicted the most favorable adsorption configurations and identified the probable site of attack of FEN on the silver surface, revealing key adsorption geometries contributing to SERS enhancement. The theoretical results were essential for assigning the vibrational modes of FEN. The combination of experimental and theoretical analyses led to the successful detection of FEN, with a remarkable limit of quantification (LOQ) of 9.9 × 10^−8^ mol/L (30 ppb). This integrated approach provides precise insights into the molecular properties and detection capabilities of FEN.

## 1. Introduction

Fenazaquin (FEN) (Figure 1) is a quinazoline-based pesticide primarily utilized as a miticide and insecticide across various crops, including pome and citrus fruits. Its mode of action involves disrupting mitochondrial function in target pests, leading to their control [1]. Numerous analytical techniques have been explored for FEN detection in diverse matrices such as soil, water, and agricultural produce. High-Performance Liquid Chromatography (HPLC) has been a standard method due to its precision and reliability [2]. Advanced techniques, such as Gas Chromatography–Mass Spectrometry (GC-MS) and Liquid Chromatography coupled with Tandem Mass Spectrometry (LC-MS/MS), have demonstrated enhanced sensitivity, enabling the detection of trace amounts of FEN [3]. Additionally, the incorporation of novel methods like QuEChERS extraction combined with switchable solvent liquid-phase microextraction has further improved the efficiency of sample preparation and detection [4].

In this work, Raman spectroscopy, surface-enhanced Raman spectroscopy (SERS) and Density Functional Theory (DFT) analysis collectively form a robust triad for investigating FEN’s detection, which contributes to advancements in environmental monitoring, food safety, and pesticide management [5]. SERS has revolutionized the landscape of molecular detection by offering exceptional sensitivity and specificity [6]. In the pursuit of effective and precise pesticide detection, the convergence of experimental and theoretical methodologies has become essential. This kind of analysis is a key point in the understanding of the most advantageous conditions for the effective adsorption of FEN on a metallic surface and the optimization of its detection by SERS.

The analysis with Raman spectroscopy offers significant benefits, particularly in delivering comprehensive structural insights, which makes it an effective technique for examining the molecular vibrations of analytes without requiring additional labeling. In this context, a Raman technique involving using plasmonic nanoparticles to enhance the signal, i.e., surface-enhanced Raman spectroscopy (SERS), is a powerful method to detect trace concentrations of pesticides in general and fungicides in particular [7]. However, no previous study has been published on the detection of FEN by SERS up to now. In SERS, the interaction between molecules and plasmon-enhanced electromagnetic fields on metal surfaces results in a significantly amplified Raman signal [8,9].

The SERS enhancement mechanism is mainly attributed to two complementary effects: electromagnetic enhancement and chemical enhancement. In this work, silver nanoparticles aggregated by KNO_3_ generate localized surface plasmon resonance (LSPR), forming highly active electromagnetic “hot spots” that significantly amplify the Raman signal [10,11,12,13]. In addition, FEN molecules adsorb onto the silver surface through active sites, enabling charge-transfer interactions between the analyte and the metal substrate, which further enhances the SERS response. The presence of Ag atomic clusters provides additional adsorption sites, promoting stronger molecule–surface interactions and contributing to signal enhancement. These combined effects play a key role in improving detection sensitivity [14,15]. The distinct advantage of SERS lies in its ability to enhance weak Raman signals, thereby improving the detection limits and facilitating the identification of FEN [16]. Unlike chromatography-based methods, which require complex instrumentation, lengthy analysis times, and extensive sample preparation, SERS enables rapid, label-free, and non-destructive detection with minimal sample volumes. Moreover, SERS provides molecular-level vibrational information, allowing for both qualitative and quantitative analysis, even at trace concentrations. These benefits, combined with its adaptability to portable devices, make SERS a compelling alternative for in-field or real-time pesticide monitoring and on-site detection in the food supply chain and environment monitoring [17].

Since the effectiveness of SERS experiments depends on the chemical affinity between the analyte and the nanoparticle surface, highlighting the critical role of surface properties in signal enhancement, several plasmonic nanoparticles can be employed to tailor these properties. To further enhance the signal intensity, the addition of salts is commonly employed to induce the aggregation of nanoparticles in suspension, thus creating localized regions of intense electric field enhancement known as “hot spots” [18]. In particular, the presence of Ag atomic clusters on the surface can create adsorption points where the adsorption and metal-to-adsorbate charge transfer could be favored, as they are complementary “hot spots” of the SERS enhancement [19].

Recent studies have demonstrated the effectiveness of concentration-dependent SERS analysis for sensitive detection. These studies showed that analyte concentration can strongly influence the SERS intensity and adsorption behavior on metallic nanostructures, providing important insights into molecule–surface interactions and enhancement mechanisms [20,21,22]. Such advances further highlight the potential of SERS as a rapid and highly sensitive technique for trace-level detection applications.

Moreover, the utilization of DFT in theoretical exploration stands as a crucial complement to experimental methodologies. DFT serves as a computational tool enabling the calculation of the essential molecular features, electronic configuration, and vibrational frequencies of FEN, eliminating the necessity for immediate experimentation [23]. DFT provides insight into the vibrational characteristics and electronic properties of the target molecule [24]. By the application of DFT, the objective in the present work was to elucidate the theoretical Raman spectra of the FEN molecule, thereby deepening our comprehension of its vibrational modes, and we also used experimental vibrational spectra to better analyze the Raman and SERS spectra [25]. Another application was the analysis of the vibrational modes of complexes of FEN with Ag clusters that could be formed onto the surface. To further understand and optimize molecular detection, Monte Carlo simulations have been employed with the aim to model and predict the most favorable binding configurations of FEN on the Ag surfaces, thus enhancing interpretation of experimental spectra [26,27].

Therefore, the novelty of the present work lies in the first reported application of SERS combined with DFT calculations and Monte Carlo simulations for the detection and vibrational characterization of FEN. The combination of experiments with theory helps us understand how FEN interacts with silver nanostructures, making it possible to detect very small amounts accurately. These results support the development of quick, non-destructive, and highly sensitive methods for monitoring pesticides, which could be useful for environmental safety and ensuring food quality.

## 2. Experimental and Theoretical Methods

### 2.1. Materials

AgNO_3_ (≥99.0%), hydrochloride hydroxylamine (99%), sodium hydroxide (≥98%), trisodium citrate trihydrate (≥99%), potassium nitrate (KNO_3_) (≥99%), ethanol (≥99.8%) and FEN were purchased from Sigma-Aldrich (analytical reagent) (St. Louis, MO, USA). All aqueous solutions used for the silver colloid synthesis were prepared using Millipore MilliQ water (Burlington, MA, USA).

### 2.2. Preparation of Ag Nanoparticles

The AgNPs obtained by reduction with hydroxylamine (AgSH) [28] were prepared by adding 300 µL of sodium hydroxide (1 M) in 90 mL of hydrochloride hydroxylamine aqueous solution (1.66 × 10^−3^ M) under stirring. Then, 10 mL of Ag nitrate aqueous solution (1 × 10^−2^ M) were added to this mixture, drop by drop. Finally, the colloidal suspension remained under stirring for 15 min and was then kept at room temperature for at least 24 h before starting the experiments. The suspension of Ag nanoparticles possessed a grayish-yellow color [29].

Citrate AgNPs (AgCT) were prepared by the following procedure. A total of 1 mL of 1% (*w*/*v*) CT aqueous solution was added to 50 mL of a boiling 10^−3^ M silver nitrate aqueous solution, and boiling was continued for 1 h. The obtained colloidal suspension showed a turbid gray aspect and had a final pH of 6.5.

### 2.3. Preparation of Samples

The Raman spectrum of the powder was registered on the solid microcrystal deposited on a quartz slide. The sample for UV-vis spectroscopy of the colloidal suspension of AgNPs was prepared by diluting 500 µL of the colloid in 2 mL of water. The UV-vis spectra were also obtained for colloidal suspensions aggregated by the addition of potassium nitrate (KNO_3_); this approach allows us to see how KNO_3_ (0.5 M) influences the spectral characteristics of AgNPs [30]. Then, 2 samples of FEN were prepared as shown in Table S1. Samples for the SERS analysis were prepared first with only 500 µL of the AgNp colloid and then by adding 20 µL of the above KNO_3_ aqueous solution for the second sample and finally by adding two aliquots of the FEN 10^−3^ M stock solution prepared in ethanol (1 µL and 0.5 µL) and two aliquots of the FEN 10^−4^ M (4 µL, and 0.5 µL) solution to 500 µL of the AgNp colloid, where 20 µL of KNO_3_ was previously added to activate the suspension. Using this method, suspensions reaching the following concentrations were prepared: 1.9 × 10^−6^ M, 9.9 × 10^−7^, 7.6 × 10^−7^ M, and 9.9 × 10^−8^ M, respectively (Table S2). For TEM analysis, the colloidal dispersion was dried on a grid following the same conditions of SERS measurements for the Ag colloid and for AgSH-KNO_3_ in the absence of FEN and in the presence of FEN.

### 2.4. Instrumentation

The UV/VIS/NIR absorbance spectra of the FEN solutions and of the colloidal dispersion were obtained with a Shimadzu 3600 UV/VIS/NIR spectrometer (Kyoto, Japan) equipped with 2H and W lamps as sources and a photomultiplier (UV/VIS), an InGaAs and a PbS (NIR) as detectors. The baseline reference sample was prepared with 2 mL of MilliQ water. UV Probe software version 2.31 was used to launch the measurements. For TEM analysis, the colloidal dispersion was dried on a grid following the same conditions of SERS measurements for the Ag colloid and for AgSH-KNO_3_ in the absence of FEN and in the presence of FEN.

Raman and SERS spectra were measured at the laboratory of the Instituto de Estructura de la Materia, Consejo Superior de Investigaciones Cientificas (IEM-CSIC), in Madrid (Spain), with a Renishaw inVia Raman spectrometer coupled with an optical microscope (Leica), equipped with Peltier-cooled CCD detector. All the Raman and SERS spectra were measured by using an excitation line, a laser at 532 nm (frequency-doubled Nd-Yag laser), using an edge filter and a 1800 L/mm grating, working at 532 nm. The spectral resolution and slit size were 2 cm^−1^ and 65 µm, respectively. An exposure time of 10 s, 10 accumulations and 10% power were used for the l × 50 objective long focal lens. Spectra were collected in the range 1800–100 cm^−1^. The software WIRE 5.6 was used to launch the acquisitions.

The TEM images were conducted at the Universidad Complutense de Madrid using a JEM 1400 PLUS JEOL (Tokyo, Japan) with a voltage in the range of 40 to 120 kV at intervals of 33 V. The TEM mode presents a resolution of 0.38 nm between points and 0.2 nm between lines, with magnification ranging from 50× to 12,105×. The camera has a focal length of 15 to 350 cm in AS DIFF and 4 to 80 cm in HD DIFF and a LaB6 filament was used to generate the electron beam.

### 2.5. DFT Calculations

Density Functional Theory (DFT) calculations were conducted using the Gaussian 16 software (Wallingford, CT, USA) [31]. The molecular structure was visualized with the Gauss View 6 (Wallingford, CT, USA) interface. Geometry optimization is a fundamental computational chemistry technique used to determine the most stable or energetically favorable conformation of a molecule. This process involves finding the point where the first derivative of the energy with respect to atomic distances, known as the gradient, is zero. At this stationary point, both the gradient and the forces acting on the atoms are null. The optimized structure of FEN was obtained by applying the DFT method with the B3LYP functional and a mixed basis set: 6-311++G(d,p) for H, C, N, and O atoms, and LANL2DZ for the silver atom [32].

### 2.6. Monte Carlo Simulation Details

Monte Carlo simulation is a well-established computational approach for investigating molecular adsorption on surfaces and evaluating the associated adsorption energies. In this study, the adsorption behavior of FEN on a silver surface was explored using the Materials Studio software suite (BIOVIA, 2020) (San Diego, CA, USA) [26]. Silver crystal structures were obtained from the Materials Studio database. Surface models were constructed by selecting the cleaving plane (111) through the Build → Surface → Cleave Surface options. To ensure structural periodicity and surface uniformity, the Symmetry function was subsequently used to generate supercells. The surface model consisted of three atomic layers. The molecular structure of FEN was optimized to its minimum energy configuration using the COMPASS II force field within the Forcite module. The same force field was employed during Monte Carlo simulations to maintain consistency [33]. These simulations were performed using the Adsorption Locator module in the 2020 release of Materials Studio. The COMPASS II force field is particularly suited for modeling adsorption phenomena, making it highly applicable in studies related to catalysis, materials engineering, and environmental systems. Through this approach, detailed insights into the interaction mechanisms and stability of FEN adsorbed on silver surfaces were obtained, contributing to a deeper understanding of its surface behavior [34].

## 3. Results and Discussion

### 3.1. Extinction Spectra of FEN

The addition of FEN to the AgSH suspension induced notable changes in the UV-Vis spectra. The addition of KNO_3_ increases the ionic strength of the medium and reduces electrostatic repulsion between nanoparticles, inducing aggregation; this is reflected in the UV-Vis spectrum by a decrease in intensity and broadening of the LSPR peak.

The UV-Vis spectra of FEN on AgSH under different conditions are shown in Figure 1 (bottom). The AgSH colloid exhibits a distinct localized surface plasmon resonance (LSPR) peak at 404 nm, resulting from the collective oscillation of conduction electrons in the silver nanoparticles, a characteristic feature of plasmonic nanostructures [5]. TEM images (Figure 1, center) show that the AgSH nanoparticles are almost spherical, consistent with the single, symmetric LSPR band at 404 nm. The changes observed upon addition of KNO_3_ or FEN (decrease and broadening of the band) are attributed to particle aggregation, visible in the TEM images, which modifies the electromagnetic coupling between neighboring nanoparticles [27].

### 3.2. TEM Images

Figure 1a shows TEM images of nanoparticles aggregated with KNO_3_ under different conditions: AgSH-KNO_3_, AgSH-KNO_3_-Fen (1.9 μM), and AgSH-KNO_3_-Fen (0.99 μM). As can be seen in Figure 1 (inset figure), the nanoparticles integrating the AgSH suspension are almost spherical and display a bimodal dispersion in size with maxima at about 40 and 60 nm in diameter, with a mean diameter of 50 nm. These images help us observe how the shape and grouping of the nanoparticles change with the addition of KNO_3_ and different amounts of FEN. TEM analysis revealed distinct morphological characteristics of the nanoparticle samples in the presence of KNO_3_. The AgSH-KNO_3_ sample exhibited significant nanoparticle aggregation, observable as clusters and chains of particles, demonstrating the salt’s influence on particle organization. The addition of FEN to the salt-containing systems (AgSH-KNO_3_-FEN at both 1.9 μM and 0.99 μM) resulted in aggregated structures, although the aggregation seems to be higher in the case of using a higher concentration of FEN, which leads to a stronger SERS intensity in the fungicide. These observations demonstrate that KNO_3_ influences the aggregation and distribution of the particles, leading to more defined and uniform clustered structures that are essential for SERS enhancement [35]. Nonetheless, other additional effects caused by nitrate are seen when analyzing the SERS spectral profiles as will be shown below.

### 3.3. Raman, SERS and DFT Spectra of FEN

The SERS spectra of FEN under different conditions are displayed in Figure 2c along with the normal Raman spectrum of the powdered FEN (Figure 2b) and the theoretical Raman spectra resulting from the DFT calculations (Figure 2a). The theoretical Raman spectrum is very similar to the experimental one, thus predicting very well the vibrational modes and providing a good assessment for the assignment of the vibrational modes. The main experimental and theoretical Raman bands are shown in Table 1. As can be seen, the Raman spectrum is a combination of three different structures existing in FEN: the quinazoline (QUZ), the tert-butylbenzene (TBB) and the linking -OCH_2_CH_2_ ethyloxi chain (ETO). The most prominent band observed in the experimental solid-state spectrum appears at 1376 cm^−1^, which is seen at 1367 cm^−1^ in the theoretical spectrum. This band is attributed to the symmetric in-plane stretching motions of the QUZ group, with a special role of the ring A (Figure 1) where the nitrogen atoms are located. These motions lead to a high modification of the electrical polarizability. The high symmetry of this motion also contributes to the high intensification of this band in the Raman spectra. In the SERS spectrum this band is moved slightly upwards to 1378 cm^−1^ due to the interaction of FEN with the metal, although, in general, the SERS spectrum does not show important modifications compared to the Raman spectra of the solid, although there are some differences that consist of a general intensification of bands attributed to the quinazoline group: those at 1579, 1547, 1024, 930, 785 and 745 cm^−1^. All these facts indicate that the QUZ group plays an important role in the interaction with Ag and it seems to be directly attached to the silver surface. The feature at 1547 cm^−1^ is not seen in the Raman spectra of the solid and it can be ascribed to the specific interaction of some of the N atoms of the quinazoline with the metal, which leads to an enhancement of this vibration upon the interaction of FEN with the metal. The changes noted in the ring-breathing region, with the appearance of two prominent bands at 785 and 745 cm^−1^, are also related to the interaction with the metal through the quinazoline group. It is interesting to note the almost disappearance of the band at 1405 cm^−1^ of the spectrum of the solid. This band is assigned to QUZ and its weakening may indicate a certain rearrangement of the structure of this group on the metal.

### 3.4. SERS of FEN on Different Metal Substrates

The SERS spectra of FEN were measured on metal nanoparticles prepared by different methods in order to find the optimal surface for the SERS detection. Figure 2 (Right) shows the spectra of the fungicide on Ag nanoparticles prepared with citrate (AgCT) (Figure 2d) and with hydroxylamine (AgSH) (Figure 2e). The SERS spectrum is much more intense on the AgSH than on AgCT probably due to the surface covering of citrate and its oxidation products after the reduction of silver. Citrate can compete with FEN in the adsorption on the surface, even after the addition of chloride to induce the possible activation of the suspension. Conversely, on AgSH the SERS intensity is ca 5 times higher [36].

The SERS spectra of FEN on AgSH are further intensified in the presence of KNO_3_ (Figure 2f). The SERS peaks observed with KNO_3_ exhibited enhanced intensity at lower concentrations of FEN, demonstrating the beneficial effect of salt addition on the detection sensitivity. This enhancement suggests that KNO_3_ promotes the formation of localized electromagnetic hot spots through partial nanoparticle aggregation and facilitates the adsorption of FEN onto the AgSH surface, even at trace levels. The chloride-functionalized surface of AgSH provides a favorable environment for FEN adsorption, while the addition of KNO_3_ further amplifies the SERS signal by optimizing the plasmonic coupling between the nanoparticles. These observations underscore the importance of surface functionalization and salt-induced aggregation in achieving sensitive and reliable SERS detection of FEN.

However, in addition to the SERS enhancement in the presence of nitrate, the FEN SERS spectra observed in the presence of the nitrate ion display evident changes in the spectral profile (Figure 2f). Specifically, new bands appear at 1522, 1339, 1193, 1070 and 783 cm^−1^. These bands are not seen in the Raman spectrum of the solid. We propose that these new bands are due to the complexation of the QUZ group with silver atoms on the metal surface, which induce a reinforcement of the interaction strength between FEN and the metal. On the other hand, the existence of Ag-to-QUZ charge transfer due to the formation of the Ag-FEN complex leads to further SERS enhancement by a chemical mechanism. In conclusion, a significant modification of the structure of FEN at the level of the QUZ moiety seems to be induced by the adsorption of the nitrate ions.

To determine the limit of detection (LOD) for FEN using this SERS-based approach, a series of experiments were conducted with varying concentrations of FEN in the presence of KNO_3_. Figure 3a shows the variation in the band at 1376 (I_1376_) versus the concentration. Figure 3b displays the variation in the Log I_1376_ versus Log[FEN]; the log–log plot (Figure 3b) is presented only as an empirical way of describing the concentration dependence of the 1376 cm^−1^ band over the wide range studied. We have considered that the non-linear response can be explained by common SERS effects derived from saturation of the active sites, steric hindrance, and the transition from monolayer to multilayer coverage and that specific intermolecular interactions may be responsible for the multilayer formation. In fact, the analysis of the spectral profile of FEN at two different concentrations: 1.9 μM (Figure 3c) and 0.76 μM (Figure 3d) reveals a change in the spectrum that can be related to this intermolecular interaction and a rearrangement on the interface. The calculation of the corresponding LOD should usually be done from the linear fitting of the I_1376_/[FEN] plot and the 3σ/S calculation, with σ being the standard deviation of the response and S the slope of the linear fitting. This is a theoretical value that is only valid when the SERS signal is linearly related to the concentration. In the present case, the response is not linear. The standard LOD formula therefore cannot be applied rigorously, and any value obtained would be an extrapolation. For this reason, the LOD was not estimated, and only the LOQ is reported. The LOQ is the lowest FEN concentration measured experimentally with a reproducible signal: LOQ = 9.9 × 10^−8^ M (about 30 ppb). However, in the present case no Langmuir-like behavior was observed; thus, estimation of the LOD was not possible. Since the LOD value is usually LOQ divided by 3–10, depending on the cases, we have estimated the LOD to be 3–10 ppb for FEN. On the other hand, the maximum residue limit (MRL) permitted by the European Union depends on the specific crop and region, but it ranges from 0.2 to 30 ppm (Regulation (EC) N0. 396/2005). Thus, the LOD and LOQ deduced from the present work clearly fulfill the needed analytical requirement to be applied in the detection of FEN in agricultural products.

The calculation of the enhancement factor (EF) for the SERS of FEN is difficult to do due to the fact that no good spectrum of the fungicide could be obtained in solution. However, an estimation of the EF can be done by comparison with the Raman spectrum of FEN in a solid state, where a concentration of 3.78 M was calculated on the basis of the compound’s density. Taking into account that the sampling conditions were equivalent for both normal Raman spectroscopy and SERS experiments in terms of probed volume, laser power, objective amplification, etc., a final EF value of 10^6^ was calculated for the AgSH nanoparticles. The AgCT colloidal suspension gave rise to a much lower EF due to the existence of citrate and its oxidation products on the surface. The calculated EF could be much lower, reaching the value of 10^7^, considering that the real number of molecules placed in good conditions for high SERS intensification, i.e., in hot spots, is actually 5–10% of the total amount of FEN molecules existing in the medium.

### 3.5. Monte Carlo Simulation Results

The interaction between the FEN molecule and the Ag (111) surface was analyzed based on the optimized adsorption geometry obtained through Monte Carlo simulations. As illustrated in Figure 4, FEN adopts a nearly planar orientation relative to the silver surface, facilitating close contact with its electron-rich functional groups. Notably, the interaction occurs through the nitrogen atoms and the carbonyl oxygen, with measured distances of 3.415 Å, 3.019 Å and 3.207 Å from the Ag surface atoms. These distances fall within the typical range for physisorption, indicating non-covalent interactions dominated by van der Waals forces and possible weak charge transfer [37]. Among these, the shortest distance (3.019 Å) suggests a stronger local interaction, as reduced separation between the molecule and the surface enhances the overlap of electron clouds, thereby increasing adsorption strength [38]. The calculated adsorption energy of FEN on the Ag (111) surface is −89.61 kJ/mol, confirming a stable and favorable adsorption process. This value is consistent with a physisorption mechanism, indicating sufficient interaction strength to ensure effective molecule anchoring while still allowing efficient surface mobility. Such behavior is beneficial for the formation of SERS-active configurations, where optimal adsorption is required to maximize signal enhancement. The adsorption process is further influenced by the presence of nitrate ions, which modify the surface charge environment and promote stronger molecule–surface interactions. Overall, the combination of favorable adsorption energy and short interaction distances supports efficient charge-transfer and electromagnetic enhancement mechanisms in surface-enhanced Raman spectroscopy (SERS), making the observed geometry highly suitable for sensitive detection applications [39].

### 3.6. DFT Calculations of the FEN-Ag10 Cluster

Figure 5 presents a comparison between the simulated Raman spectrum of the FEN-Ag_10_ complex and the experimental SERS spectrum of FEN adsorbed on a silver substrate in the presence of nitrate. As can be seen, the model reveals that the interaction of the Ag_10_ cluster occurs at the level of the N1 atom in the QUZ group. In addition, there is high agreement between the theoretical and experimental spectra, i.e., the SERS in the presence of nitrate demonstrates that the presence of nitrate induces a strong interaction between the molecule and the metallic surface through the quinoleine group. Notably, a prominent enhancement at 1376 cm^−1^ in the SERS spectrum corresponds to a highly active vibrational mode related to the ν(C-N) + δ(C-C) of the QUZ group. Additional enhanced bands observed at 771, 931, 1108, 1191, 1301, 1513 and 1577 cm^−1^, mainly assigned to the QUZ group, indicate that FEN interacts with the Ag10 cluster through the N1 atom of the quinazoline group. The slight differences between the theoretical and experimental peak positions further support the occurrence of adsorption-induced structural or electronic changes [40] due to the formation of the complex. These results confirm effective adsorption of FEN onto the silver nanocluster, suggesting a chemisorption mechanism facilitated by charge transfer induced by the presence of silver clusters existing in the surface of the Ag nanoparticles, which underlies the observed SERS signal enhancement [41]. The similarities between the theoretical spectrum of the FEN-Ag_10_ cluster and the experimental SERS registered in the presence of nitrate corroborate the increased importance of the charge-transfer effect in the SERS enhancement of FEN in the presence of the anion and account for the enhancing contribution of the chemical effect in the sensitivity increase.

## 4. Conclusions

In conclusion, this study highlights the successful application of surface-enhanced Raman spectroscopy (SERS) for the ultrasensitive detection of FEN, achieving a notably low limit of quantification (LOQ) of 30 ppb, which is comparable to that obtained with other chromatographic methods. The use of optimized silver nanoparticle substrates, with a combination of chloride in AgSH colloidal suspension and potassium nitrate as an activator, seems to create the needed atomic clusters on the metal surface to further enhance the SERS intensification by a combination of electromagnetic and charge-transfer effects. The theoretical modeling through Density Functional Theory and Monte Carlo simulations provided crucial insight into the adsorption behavior and vibrational characteristics of the molecule, both in isolation and in the interaction with planar surfaces and Ag_10_ clusters. The models confirm the importance of the electromagnetic model contribution to the SERS enhancement as well as the charge transfer provided by the metal clusters existing on the silver surface. Finally, the strong correlation between the experimental and computational results confirms the reliability of this integrated approach, demonstrating its effectiveness for trace-level pesticide detection and its potential for broader environmental monitoring applications.

## Figures and Tables

**Figure 1:**
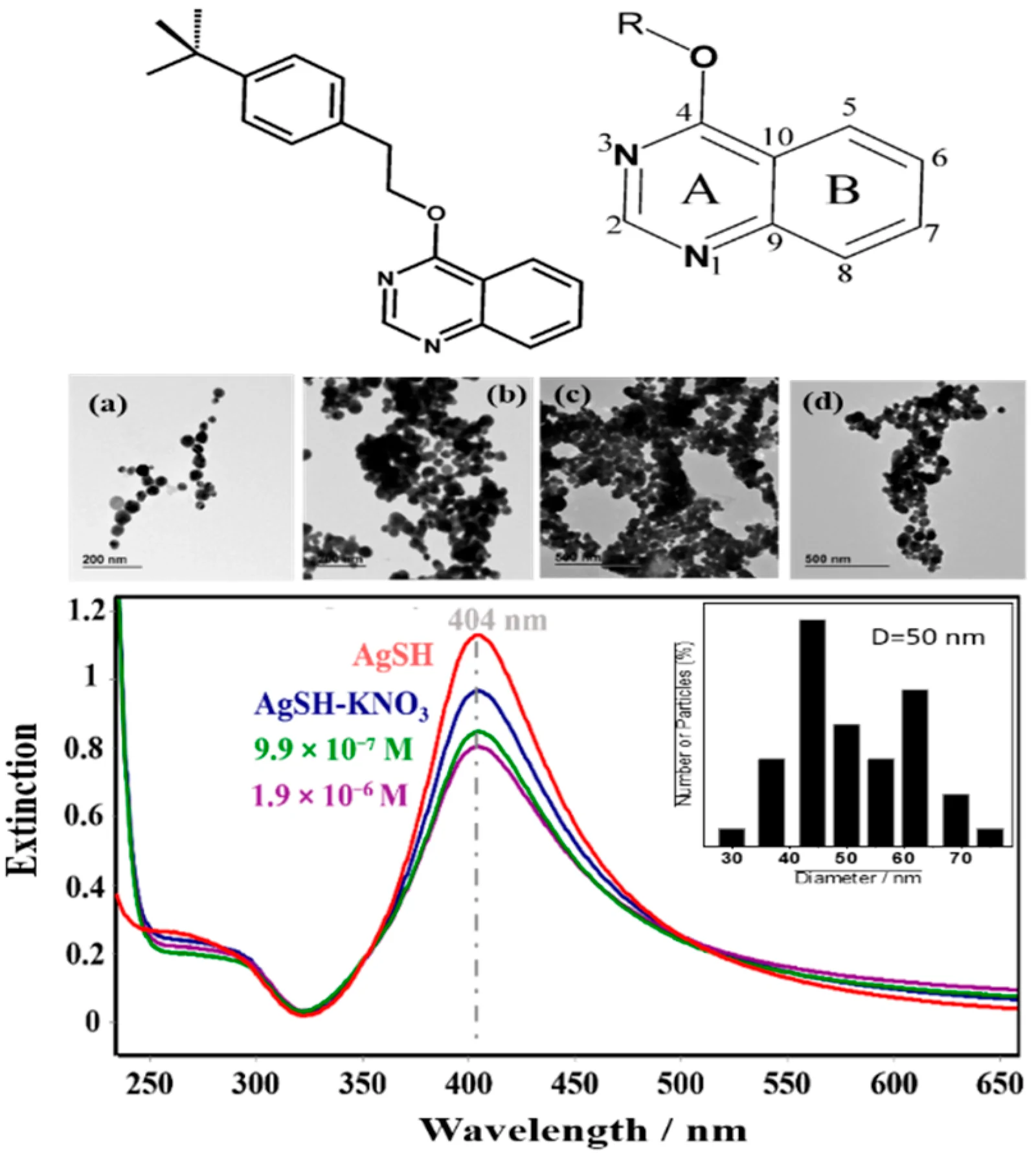
Top: Chemical structure and atom numbering of QUZ group in FEN. Center: TEM images of AgSH (**a**), AgSH aggregated with KNO_3_ (**b**), and AgSH with FEN at concentrations of 1.9 and 0.99 μM ((**c**) and (**d**), respectively) previously aggregated with KNO_3_ (20 mM). Bottom: Extinction spectra of FEN (at different concentrations as indicated in each spectrum) on AgSH nanoparticles after aggregating with KNO_3_ (20 mM). Inset figure: Dispersion of nanoparticle sizes in the analyzed TEM images.

**Figure 2:**
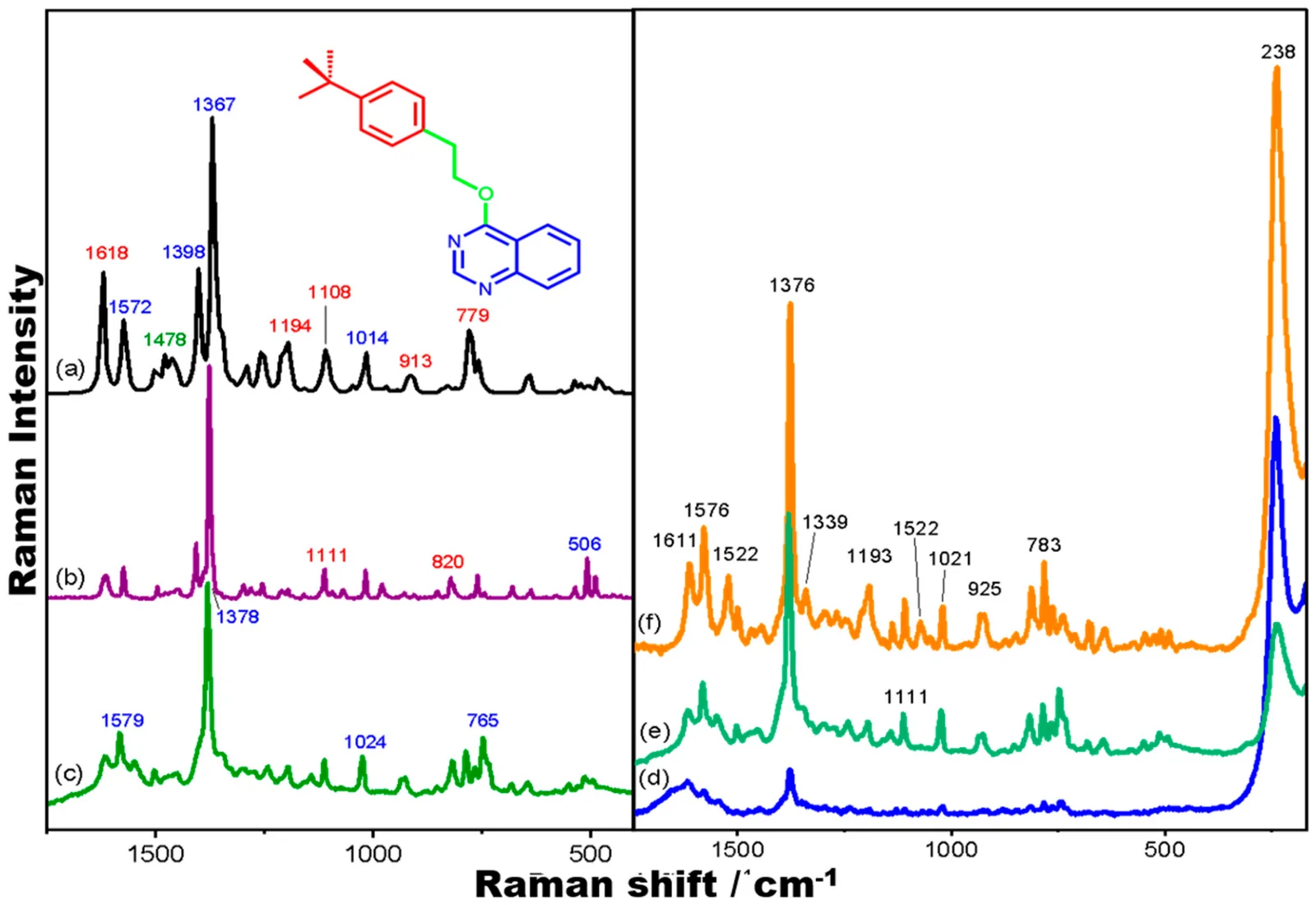
Left: Theoretical Raman spectrum calculated by DFT methods (**a**); Raman spectrum of solid FEN (**b**) and SERS spectrum of FEN (1.9 μM) in AgSH nanoparticles without potassium nitrate (**c**). Right: SERS spectra of FEN (1.9 μM) in different nanoparticles: AgCT (**d**); AgSH (**e**); and AgSH with FEN at a concentration of 1.9 μM aggregated previously with KNO_3_ (20 mM) (**f**). Excitation laser at 532 nm.

**Figure 3:**
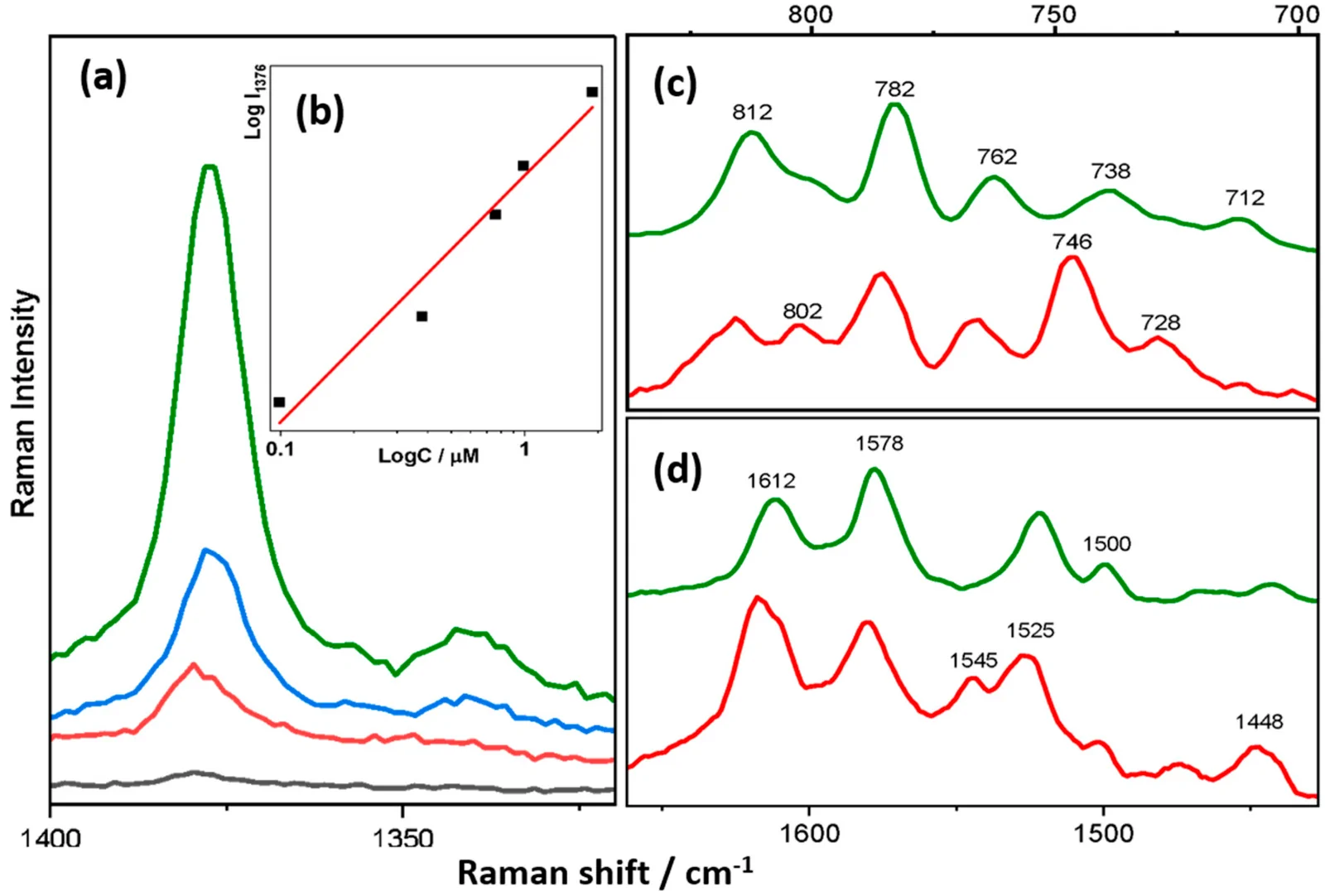
Variation in the 1356 cm^−1^ band in the SERS spectra of FEN on AgSH nanoparticles aggregated previously with KNO_3_ (20 mM) at different concentrations (**a**). (**b**): Plot of Log I_1376_ vs. Log[FEN]. (**c**,**d**) Comparison between the spectral profiles of the SERS spectra of FEN at concentrations of 1.9 μM (green) and 0.76 μM (red) for two different spectral regions. Excitation laser at 532 nm.

**Figure 4:**
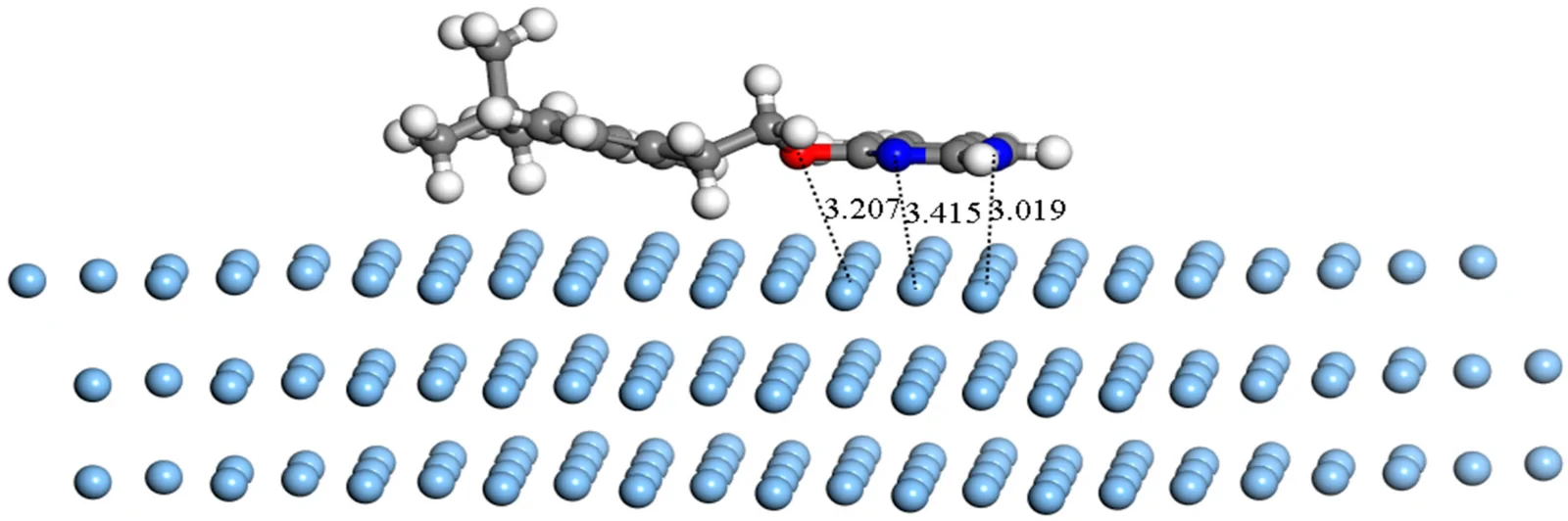
Optimized structure of FEN adsorbed on the Ag (111) surface.

**Figure 5:**
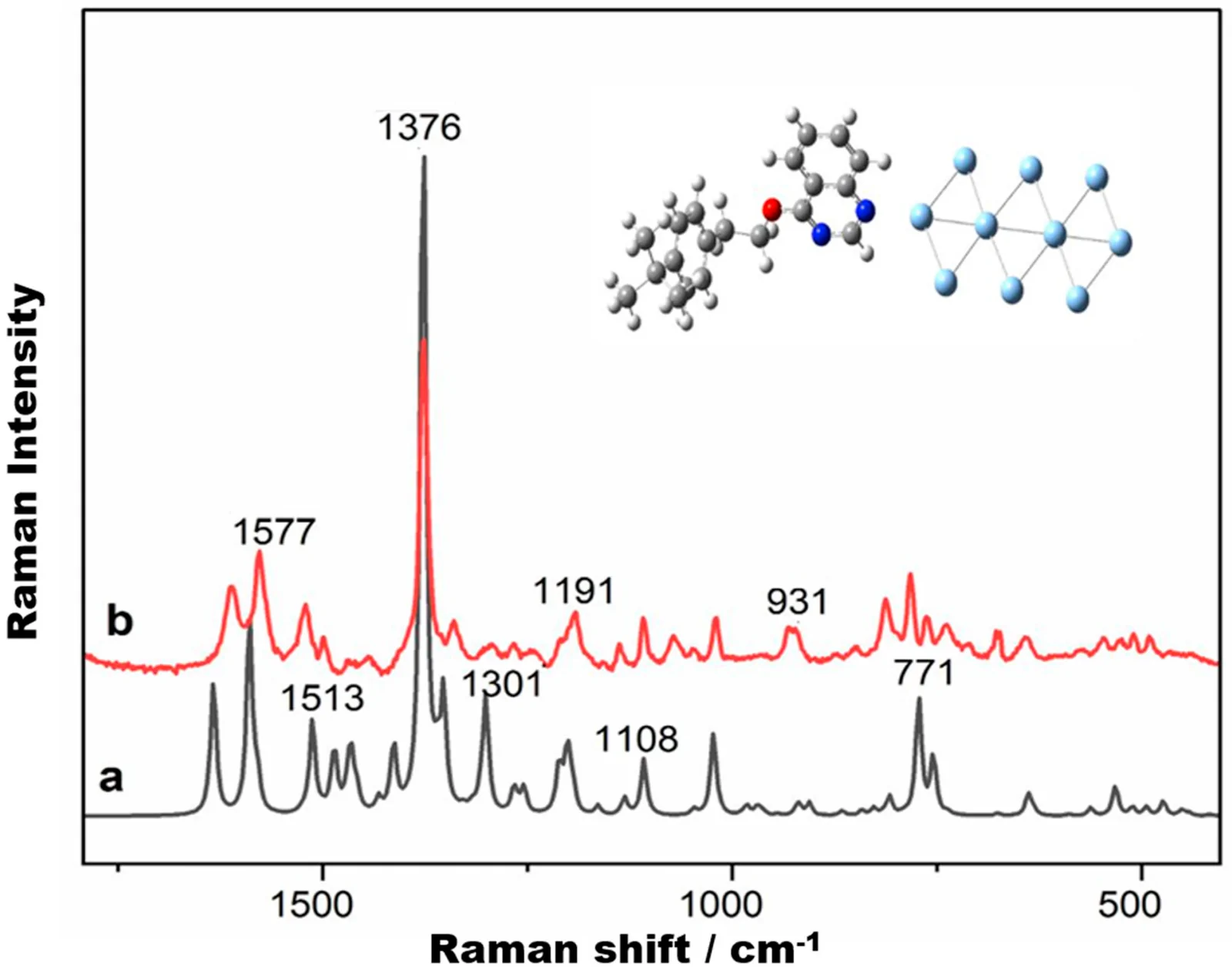
Simulated Fen-Ag_10_ (**a**) and experimental SERS spectra (**b**) of FEN (1.9 μM) adsorbed on a silver substrate.

**Table 1:** Main wavenumbers and assignments of the experimental and theoretical Raman and SERS spectra of FEN.

Raman FEN Solid	DFT Isolated FEN	SERS (AgSH)/KNO_3_ (1.9 μM)	DFT of FEN- Ag10	Assignments *
506	504	511	533	δ(CCC)/δ(CNC) in QUZ
637	640	643	637	δ(CCC)/δ(CNC) in QUZ
677		675		δ(CCC) in TBB and QUZ
742		745	755	RB(QUZ)
759	756	736		RB(QUZ)
775	779	783	771	RB(TBB)
820	827	812	807	RB(TBB)
927	913	925	918	ν(CC) in TB
977	969		967	ν(CC) in ETO
1016	1014	1021	1023	RB(Ring B in QUZ)
1111	1108	1109	1108	ν(CBenzene-CTB)
1193	1194	1193	1199	ν(CC) in QUZ/ν(CC) in TB/δ(CH)
1252	1254	1243	1256	ν(CC) in QUZ/δ(CH)
1297	1290	1295	1301	ν(CC) in QUZ/ν(CO) ω(CH_2_) in ETO
		1339	1353	ν(CC) in QUZ/ν(CO)
1376	1367	1376	1376	ν (CN) and ν(C-C) in QUZ
1405	1398		1412	ν (CN) and ν(C-C) in QUZ/δ(CH)
1450	1460	1441	1464	δ(CH_2_)/δ(CH_3_)
1492	1499	1499	1487	δ(CH_2_)/δ(CH_3_)
		1522	1513	ν(CN) and ν(C-C) in QUZ-Ag complex
1572	1572	1577	1588	ν(CN) and ν(C-C) in QUZ
1613	1618	1611	1634	ν(CN) and ν(C-C) in TBB

* δ: bending; ν: stretching; ω: wagging; RB: ring-breathing mode; QUZ: quinazoline; TBB: tert-butyl benzene; ETO: ethyloxi; TB: tert-butyl.

## Data Availability

The datasets used and/or analyzed during the current study are available upon request.

## Supplementary Materials

The following supporting information can be downloaded at https://www.mdpi.com/article/10.3390/molecules31214009/s1, Table S1: Preparation of the samples (Ag colloid + KNO_3_ + Fenazaquin) used in the extinction measurements; Table S2: Preparation of the samples (Ag colloid + KNO_3_ + Fenazaquin) used in the SERS measurements. Figure S1. TEM images of AgSH, AgSH aggregated with KNO3, and AgSH with FEN.
