# Supplementary material for: Experimental and Computational Insights into SERS-Based Ultrasensitive Detection of the Pesticide Fenazaquin: Towards Food Safety and Environmental Analysis
Source: Molecules. 2026 Nov 6;31(21):4009. doi: 10.3390/molecules31214009 (PMC13765008; doi:10.3390/molecules31214009)
Supplement: Supplementary file 1 [file molecules-31-04009-s001.zip › molecules-4636464-supplementary.pdf]

Supporting Information

Experimental and Computational Insights into SERS-Based Ultrasensitive Detection of the pesticide Fenazaquin: Towards Food Safety and Environmental Analysis

Oumaima Douass[1,2*],*Ibrahim Saadouni*[1],*Bousselham Samoudi*[1] , *Ana Crespo*[2] and Santiago *Sanchez-Cortes*[2]

[1] *Optics, Material and Systems Team, Faculty of Sciences, Abdelmalek Essâadi University, B.P. 2121, M' Hannech II, 93030 Tétouan, Morocco.*

[2] *Instituto de Estructura de la Materia, IEM-CSIC, 28006 Madrid, Spain.*

***Corresponding author**

E-mail address: oumaima.douass@etu.uae.ac.ma

Quantitative analysis – sample preparation

Table S1. Preparation of the samples (Ag colloid+KNO_3+ Fenazaquin) used in the extinction measurements.

**Stock solution (mol/L)**	**Volume added (µL)**	**Total volume of the sample (µL)**	**Concentration in the sample (mol/L)**
$1.0x10^{-3}$	0.5	520.5	$9,9\times10^{-7}$
	1	521	$1,9\times10^{-7}$

* The stock solution was prepared, and the calculation was taken for an initial volume of 500 µL of Ag colloid and 20 µL of KNO_3. Subsequently, the volume of Fenazaquin was added, along with an additional 2000 µL of ultrapure water in the final composition for each sample.

Table S2. Preparation of the samples (Ag colloid+KNO_3+Fenazaquin) used in the SERS measurements.

**Stock solution (mol/L)**	**Volume added (µL)**	**Total volume of the sample (µL)**	**Concentration in the sample (mol/L)**
$1.0x10^{-3}$	0.5	520.5	$9,9\times10^{-7}$
	1	521	$1,9\times10^{-7}$
$1.0x10^{-4}$	0.5	520.5	9.9×10^{-8}
	4	524	7.6×10^{-7}

* Two stock solutions were prepared, with each calculation based on an initial volume of 500 µL of Ag colloid and 20 µL of KNO_3. Subsequently, the volume of Fenazaquin was added in the final composition for each sample.

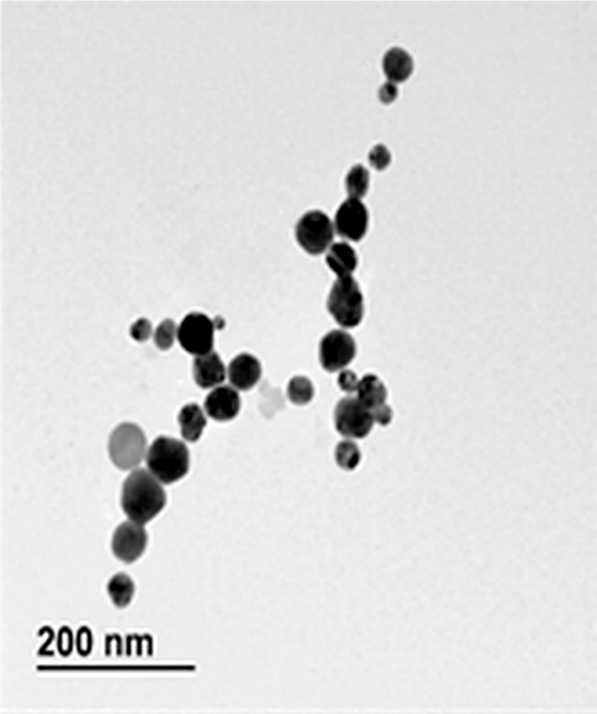

AgSH

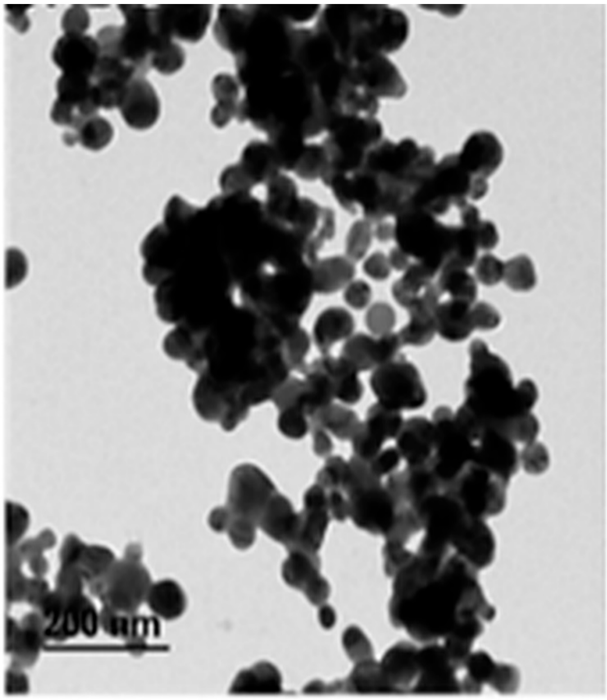

AgSH-KNO_3

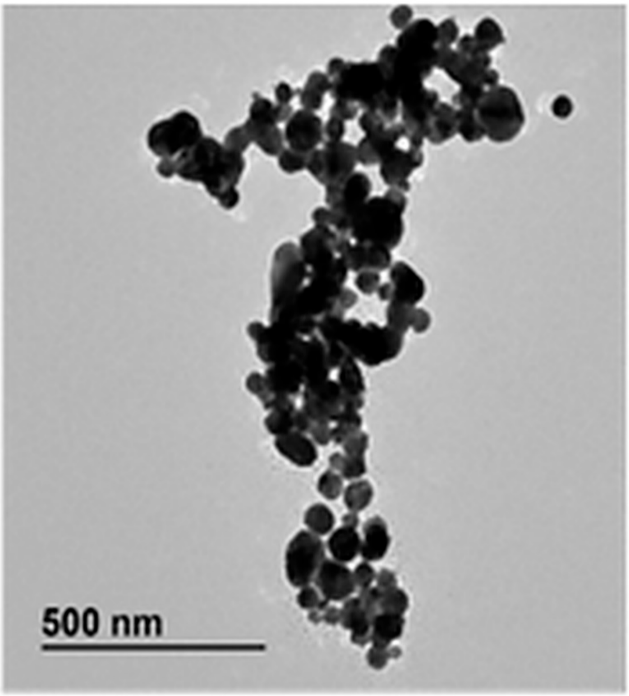

AgSH-KNO_3-Fen(9.9×10^{-7})

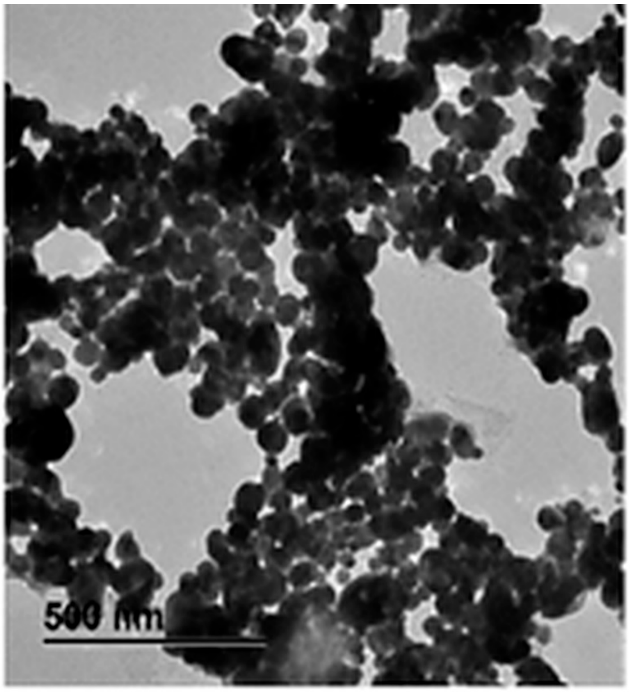

AgSH-KNO_3-Fen(1.9×10^{-6})

Figure S1. TEM images of AgSH , AgSH aggregated with KNO_3, and AgSH with FEN.
